# CONDUCTING DIVERSITY, EQUITY, AND INCLUSION-INFORMED RESEARCH WITH OLDER ADULTS

**DOI:** 10.1093/geroni/igac059.636

**Published:** 2022-12-20

**Authors:** Ashley Taeckens-Seabaugh

**Affiliations:** University of Denver, Denver, Colorado, United States

## Abstract

Researchers are encouraged to think broadly and holistically about how they conceptualize diversity, equity, and inclusion (DEI) efforts in their work. Each element of a research project must be rigorously devised to identify or address existing gaps in the literature, which will continue to be increasingly important as the US population of older adults continues to expand and diversify. Intentionality throughout every stage of a project is bound to result in a better understanding of the focus populations’ needs, and how to best address those needs through their perspective. Moreover, consider weighing the pros and cons of working to implement culturally competent versus multiculturally competent research and whether either approach is as culturally appropriate and responsive as possible. This work summarizes guidance to increase DEI efforts by meticulous research question development and thoughtful determinations regarding literature review terminology, recruitment efforts, data collection, and reporting and dissemination techniques.

